# Artificial intelligence to improve ischemia prediction in Rubidium Positron Emission Tomography—a validation study

**DOI:** 10.1007/s13167-023-00341-5

**Published:** 2023-11-15

**Authors:** Simon M. Frey, Adam Bakula, Andrew Tsirkin, Vasily Vasilchenko, Peter Ruff, Caroline Oehri, Melissa Fee Amrein, Gabrielle Huré, Klara Rumora, Ibrahim Schäfer, Federico Caobelli, Philip Haaf, Christian E. Mueller, Bjoern Andrew Remppis, Hans-Peter Brunner-La Rocca, Michael J. Zellweger

**Affiliations:** 1 Department of Cardiology, University Hospital Basel, University of Basel, Petersgraben 4, CH-4031 Basel, Switzerland; 2 Cardiovascular Research Institute Basel (CRIB), University Hospital Basel, University of Basel, Spitalstrasse 2, CH-4056 Basel, Switzerland; 3 University Hospital Bern, University of Bern, Freiburgstrasse 18, CH-3010 Bern, Switzerland; 4 Exploris Health AG, Industriestrasse 44, CH-8304 Wallisellen, Switzerland; 5 Department of Cardiology, Herz- Und Gefässzentrum Bad Bevensen, Römstedter Straße 25, 29549 Bad Bevensen, Germany; 6 Department of Cardiology, Maastricht UMC+, Minderbroedersberg 4-6, Maastricht, 6211 LK The Netherlands

**Keywords:** Coronary artery disease (CAD), Pretest probability (PTP), Patient stratification, Risk stratification, Ischemia, Positron emission tomography (PET), Artificial intelligence, Predictive preventive personalised medicine (PPPM/3PM), Gatekeeper, Improved individual outcome

## Abstract

**Background:**

Patients are referred to functional coronary artery disease (CAD) testing based on their pre-test probability (PTP) to search for myocardial ischemia. The recommended prediction tools incorporate three variables (symptoms, age, sex) and are easy to use, but have a limited diagnostic accuracy. Hence, a substantial proportion of non-invasive functional tests reveal no myocardial ischemia, leading to unnecessary radiation exposure and costs. Therefore, preselection of patients before ischemia testing needs to be improved using a more predictive and personalised approach.

**Aims:**

Using multiple variables (symptoms, vitals, ECG, biomarkers), artificial intelligence–based tools can provide a detailed and individualised profile of each patient. This could improve PTP assessment and provide a more personalised diagnostic approach in the framework of predictive, preventive and personalised medicine (PPPM).

**Methods:**

Consecutive patients (*n* = 2417) referred for Rubidium-82 positron emission tomography were evaluated. PTP was calculated using the ESC 2013/2019 and ACC 2012/2021 guidelines, and a memetic pattern–based algorithm (MPA) was applied incorporating symptoms, vitals, ECG and biomarkers. Five PTP categories from very low to very high PTP were defined (i.e., < 5%, 5–15%, 15–50%, 50–85%, > 85%). Ischemia was defined as summed difference score (SDS) ≥ 2.

**Results:**

Ischemia was present in 37.1%. The MPA model was most accurate to predict ischemia (AUC: 0.758, *p* < 0.001 compared to ESC 2013, 0.661; ESC 2019, 0.673; ACC 2012, 0.585; ACC 2021, 0.667). Using the < 5% threshold, the MPA’s sensitivity and negative predictive value to rule out ischemia were 99.1% and 96.4%, respectively. The model allocated patients more evenly across PTP categories, reduced the proportion of patients in the intermediate (15–85%) range by 29% (ACC 2012)–51% (ESC 2019), and was the only tool to correctly predict ischemia prevalence in the very low PTP category.

**Conclusion:**

The MPA model enhanced ischemia testing according to the PPPM framework:

1. The MPA model improved individual prediction of ischemia significantly and could safely exclude ischemia based on readily available variables without advanced testing (“predictive”).
2. It reduced the proportion of patients in the intermediate PTP range. Therefore, it could be used as a gatekeeper to prevent patients from further unnecessary downstream testing, radiation exposure and costs (“preventive”).
3. Consequently, the MPA model could transform ischemia testing towards a more personalised diagnostic algorithm (“personalised”).

**Supplementary Information:**

The online version contains supplementary material available at 10.1007/s13167-023-00341-5.

## Introduction

Coronary artery disease (CAD) is frequent and accounts for significant morbidity, mortality and health care costs [1]. Multiple tests are available for diagnosis and risk stratification, but they are either not sufficiently accurate, invasive in nature and/or expensive. The prevalence of CAD/myocardial ischemia in patients referred for testing declined over the last decades. Hence, low-risk test results have consequently increased from around 30 to 80% between 1992 and 2012 [2]. Consequently, the proportion of normal test results is often reported to be around 60–70% which may lead to unnecessary radiation exposure for patients and high health care costs [3–5]. Given the large number of patients who need testing opposed to limited resources and potential risks of individual tests [6], only selected patients should be referred for specific advanced testing and an personalised preselection prior to testing is becoming more important.

### Current preselection tools

As recommended by the current guidelines, patients with suspected CAD are referred for further testing depending on their individual pre-test probability (PTP). Since PTP significantly affects the chosen test’s performance, it is advised to use PTP tools prior to referral [7–10]. European and American Cardiology Societies recommend in their current guidelines to estimate the PTP of CAD applying three basic variables (symptoms, age and sex) in easy-to-use tables [8, 9].

Until 2021, the American guidelines recommended to use data from the historic landmark study from Diamond-Forrester (DF) in 1979 [11]. Since PTP with DF tended to overestimate prevalence, especially in women, Genders et al. updated and recalibrated the score in 2011 [12]. This formed the basis for a score included in the ESC 2013 guidelines on chronic coronary syndromes [10]. In the latest guidelines (ESC 2019 and ACC 2021), PTP estimation is now based on the CAD prevalence of contemporary, predominantly CT coronary angiography cohorts [13].

Despite accounting for the lower prevalence of CAD in patients tested nowadays, these tables do not offer PTP calculation above 52%. Hence, all patients with a PTP ≥ 15% should be tested non-invasively and a direct referral to invasive angiogram is not intended based on the PTP. Therefore, these tools are not helpful to reduce unnecessary tests and identify patients who could be deferred from functional testing.

Despite the recommended PTP tables’ ease of use, three variables cannot sufficiently assess an individual patient because they do not comprehensively incorporate variables from different patient domains such as vitals, ECG and biomarkers. Consequently, these tools are of limited value to preselect patients before advanced cardiac testing.

### Artificial intelligence to improve preselection of patients in a PPPM framework

Hence, there is a need to improve patient selection towards a more predictive, preventive and personalised medicine (PPPM) [14]. Instead of the “one fits all” concept of these easy-to-use tools, novel models using artificial intelligence (AI) can incorporate widely and easily available variables and account for non-linear relationships and higher-order interactions between variables [15]. The factors in the AI models are not seen as independent individual values, but are recognised as patterns derived by a combinatorial analysis of the individual profile of each patient. Hence, it is not surprising that such models exceed traditional PTP tools [16–18]. Data on AI tools to predict ischemia in comparison to PTP tools are scarce [18, 19]. These tools might improve individual PTP assessment further in the direction of PPPM. However, sufficient clinical validation is often missing for such models.

### Working hypothesis

Our group has developed and validated a memetic pattern–based algorithm (MPA)–based artificial intelligence tool to detect CAD as defined by invasive angiogram [16, 17, 20]. However, in the post-ISCHEMIA trial era [21], detection and prediction of ischemia is gaining more and more importance compared to isolated anatomical description of luminal narrowing. Our approach has not yet been tested and validated to detect ischemia.

Hence, the aims of this study were to examine whether this novel AI approach excels the existing, state-of-the-art PTP scores of CAD for patient preselection and to validate this approach for the prediction of ischemia in patients referred for non-invasive testing.

If this tool worked, it could *exclude* (or predict) ischemia for an individual patient based on readily available variables. It could improve patient preselection (who needs further testing and who not) and thus *prevent* certain patients from unnecessary radiation exposure. Implemented in clinical routine, it could improve *personalization of medical services* by optimizing and individualising preselection of patients and triaging them to the test they ideally need.

## Methods

### Study design and patient selection

Consecutive patients referred for a Rubidium-82 positron emission tomography (PET) scan at a tertiary centre (University Hospital Basel) between July 2018 and February 2022 were identified and invited to participate in this prospective cohort study. If patients consented for the use of their clinical data and an additional blood sample, they were included for this project (*n* = 2417). The study flow is illustrated in Figure S1.

Baseline characteristics (cardiovascular risk factors, vital signs, ECG, medication) were collected from a detailed questionnaire filled out by the physician in charge. The study was carried out according to the principles of the Declaration of Helsinki and was approved by the local ethics committee (Ethikkommission der Nordwest- und Zentralschweiz EKNZ, ethics committee of north western and central Switzerland, project ID: PB_2018-00076/EK 67/08).

A literature search on PUBMED using the items “artificial intelligence”, “ischemia”, “prediction”, “patient stratification”, “PET” and “pretest probability” was performed with the AND function.

### Imaging protocol and analysis

Imaging protocols were used as described before [4, 22]. In short, patients were instructed to withhold caffeine-containing products for 24 h before the test. For the PET study, a 3D-PET/CT scanner was used (Biograph mCT, Siemens Healthineers, Erlangen, Germany). A low-dose CT scan was obtained for attenuation correction (increment 0.6 mm, soft-tissue reconstruction kernel, 120 keV, CAREDOSE 4D).

Thereafter, ^82^Rb was intravenously injected in a weight-adjusted manner for rest and stress images (< 100 kg: 1110 MBq (30 mCi), ≥ 100 kg 1480 MBq (40 mCi)). Rest was always performed first. After resting imaging acquisition, patients were pharmacologically stressed with adenosine (140 µg/kg/min for 6 min). If contraindications (mostly allergic asthma) or personal preferences were present, Regadenoson was used instead (400 µg single-dose). Patients were monitored according to current guidelines [23].

Dynamic, ECG-gated PET images were recorded for rest and stress over 7 min in list mode starting with tracer injection and then reconstructed as described in the supplement. ECG-gated images were analysed using QGS-QPS software included in the SyngoVia package (Siemens).

Images were analysed and interpreted by an experienced board-certified nuclear medicine physician and cardiologist as a joint read reaching consensus. A visual semi-quantitative 17-segment model with a 5-point scale (0, normal tracer uptake; 4, no tracer uptake) was used to calculate summed stress (SSS), rest (SRS) and difference score (SDS = SSS − SRS). An SDS ≥ 2 was considered threshold for ischemia.

### Calculation of pre-test probability

As published in the corresponding guidelines (ACC 2012^7^, ESC 2013^10^, ACC 2021^8^, ESC 2019^9^), the respective proposed tables were used to calculate PTP based on the available clinical information (symptoms, age and sex).

Subsequently, the memetic pattern–based algorithm (MPA) was compared against the abovementioned PTP scores. With the available clinical data, laboratory and ECG, this software tool calculates the probability of having CAD using the MPA. This multilayer non-linear complex classifier was derived from an evolutionary learning optimisation process using and combining optimal parameterisation of different methods including pattern recognition and machine learning. Initially developed in the BASEL study [20], it was further validated in a high-risk (LURIC [17]) and a low-to-intermediate risk cohort [16].

The model includes the following variables: age, sex, weight, height, presence and type of chest pain, diabetes, nicotine use, pathological Q-waves on ECG, systolic and diastolic blood pressure, relevant medication (like statin use), and biomarkers—mean corpuscular haemoglobin concentration, white blood cells, urea, uric acid, high-sensitivity cardiac troponin T, glucose, total cholesterol, low-density lipoprotein cholesterol, high-density lipoprotein cholesterol, alanine aminotransferase, alkaline phosphatase, amylase, total protein, albumin and bilirubin.

Based on these variables, the MPA model provides a numerical value between 0 and 100, which does not directly translate into PTP. The value is then used to allocate patients to one out of five PTP categories (very low to very high PTP). The calibration strongly depends on the setting (expected prevalence of CAD) in which the model is used. For this publication, we used the original calibration derived from the first external validation (LURIC [17]), and compared it also to the low-risk model [16]. PTP categories were defined as described in Table 1.

**Table 1:**
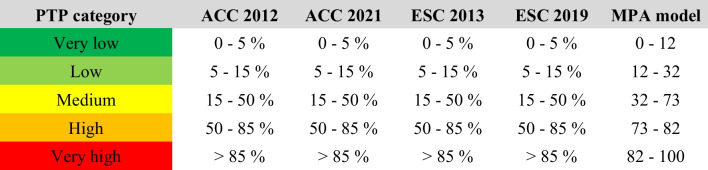
Definition of pre-test probability categories according to expected prevalence of CAD

The table shows the commonly used definition of pre-test probability (PTP) category used for the ACC and ESC guideline derived scores. The calibration of the MPA model is based on the original external validation. [17] (which is described in more detail in the “Methods” section)

### Statistical analysis

Normally distributed continuous variables are reported as mean ± standard deviation (SD) and statistical testing was performed with unpaired *t*-test or ANOVA. Categorical variables are displayed using frequencies and percentages and were compared using the chi-square test or Fisher’s exact text where appropriate. A *p*-value < 0.05 was considered as statistically significant.

Endpoint was defined as ischemia (SDS ≥ 2). Sensitivity, specificity, positive and negative predictive value (PPV, NPV), positive and negative likelihood ratio (PLR, NLR), diagnostic odds ratio (DOR) and false negative as well as false positive rate (FNR, FPR) were calculated. Receiver operating characteristic (ROC) analysis was performed to determine the area under the curve (AUC). Comparison between MPA model and other scores was performed using the DeLong method. For this calculation, a Bonferroni corrected *p*-value of < 0.0125 (α = 0.05/4, given 4 comparisons) was considered significant.

Statistical analyses were performed using SPSS™ (version 28.0.1.0) and RStudio (using R version 4.1.2).

## Results

### Patient population

A total of 2417 patients were included for this study. Mean age was 66 ± 11 years and 32% were female. Typical and atypical angina were reported in 21% and 23%, respectively. According to the four common PTP scores to assess the pre-test probability of CAD, the majority of patients had a predicted prevalence of CAD in the intermediate range of 15–85% (ACC 2012, 62.7%; ESC 2019, 65.5%; ACC 2012, 81.5%; ESC 2013, 90.2%). A total of 1120 (46.3%) patients had known CAD. Ischemia was present in 897 (37.1%) patients. More detailed baseline characteristics of the patients are displayed in Table 2.

**Table 2:** Baseline characteristics

Variable	All patients	Male	Female	*p*-value
	*n* = 2417	*n* = 1653	*n* = 764	
Age	66.1 (10.9)	65.6 (10.8)	67.1 (11.0)	0.001
BMI [kg/m^2^]	28.0 (5.3)	28.1 (4.8)	27.8 (6.2)	0.162
Q wave on ECG (%)	276 (11.4)	226 (13.7)	50 (6.5)	< 0.001
Systolic blood pressure	125.4 (21.0)	122.9 (20.1)	130.8 (21.8)	< 0.001
Diastolic blood pressure	69.5 (12.3)	68.8 (12.0)	71.1 (12.9)	< 0.001
Symptoms (%)				< 0.001
Asymptomatic	1089 (45.1)	821 (49.7)	268 (35.1)	
Non-cardiac	269 (11.1)	165 (10.0)	104 (13.6)	
Atypical angina	549 (22.7)	338 (20.4)	211 (27.6)	
Typical angina	510 (21.1)	329 (19.9)	181 (23.7)	
Known CAD (%)	1120 (46.3)	921 (55.7)	199 (26.0)	< 0.001
Prior myocardial infarction (%)	736 (30.5)	611 (37.0)	125 (16.4)	< 0.001
Prior CABG (%)	314 (13.0)	273 (16.5)	41 (5.4)	< 0.001
Prior PCI (%)	890 (36.8)	729 (44.1)	161 (21.1)	< 0.001
Risk factors				
Arterial hypertension (%)	866 (35.8)	609 (36.8)	257 (33.6)	0.138
Hypercholesterolemia (%)	804 (33.3)	581 (35.1)	223 (29.2)	0.004
Diabetes (%)	585 (24.2%)	447 (27.0%)	138 (18.1%)	< 0.001
Smoker (%)	1468 (60.7)	1110 (67.2)	358 (46.9)	< 0.001
Family history (%)	240 (9.9)	161 (9.7)	79 (10.3)	0.7
Medication				
Platelet inhibitor (%)	1404 (58.1)	1058 (64.0)	346 (45.3)	< 0.001
Antihypertensive medication (%)	1525 (63.1)	1118 (67.6)	407 (53.3)	< 0.001
Betablocker (%)	1199 (49.6)	903 (54.6)	296 (38.7)	< 0.001
Entresto (%)	39 (1.6)	32 (1.9)	7 (0.9)	0.094
ACE inhibitor (%)	372 (15.4)	283 (17.1)	89 (11.6)	0.001
AT2 blocker (%)	334 (13.8)	227 (13.7)	107 (14.0)	0.907
Lipid-lowering therapy (%)	1568 (64.9)	1180 (71.4)	388 (50.8)	< 0.001
Statin (%)	1551 (64.2)	1173 (71.0)	378 (49.5)	< 0.001
Ezetimib (%)	187 (7.7)	148 (9.0)	39 (5.1)	0.001
PCSK9 inhibitor (%)	10 (0.4)	5 (0.3)	5 (0.7)	0.361
Amiodarone (%)	19 (0.8)	17 (1.0)	2 (0.3)	0.082
Diuretic (%)	781 (32.3)	553 (33.5)	228 (29.8)	0.086
Nitroglycerin (%)	127 (5.3)	91 (5.5)	36 (4.7)	0.475
Risk scores				
ACC 2012	44.1 (29.3)	47.1 (28.8)	37.7 (29.2)	< 0.001
ACC 2021	28.8 (12.9)	34.2 (11.2)	16.9 (6.8)	< 0.001
ESC 2013	48.4 (21.1)	55.8 (17.8)	32.5 (18.8)	< 0.001
ESC 2019	20.9 (12.2)	25.0 (11.8)	12.0 (7.2)	< 0.001

Table showing baseline characteristics of included patients stratified by sex. Values are displayed as mean (SD) or frequency (percentage). ANOVA and chi-square tests were used where appropriate. *BMI* body mass index, *CABG* coronary artery bypass graft, *CAD* coronary artery disease, *PCI* percutaneous coronary intervention

### Test performance of different pre-test probability tools

**Fig. 1:**
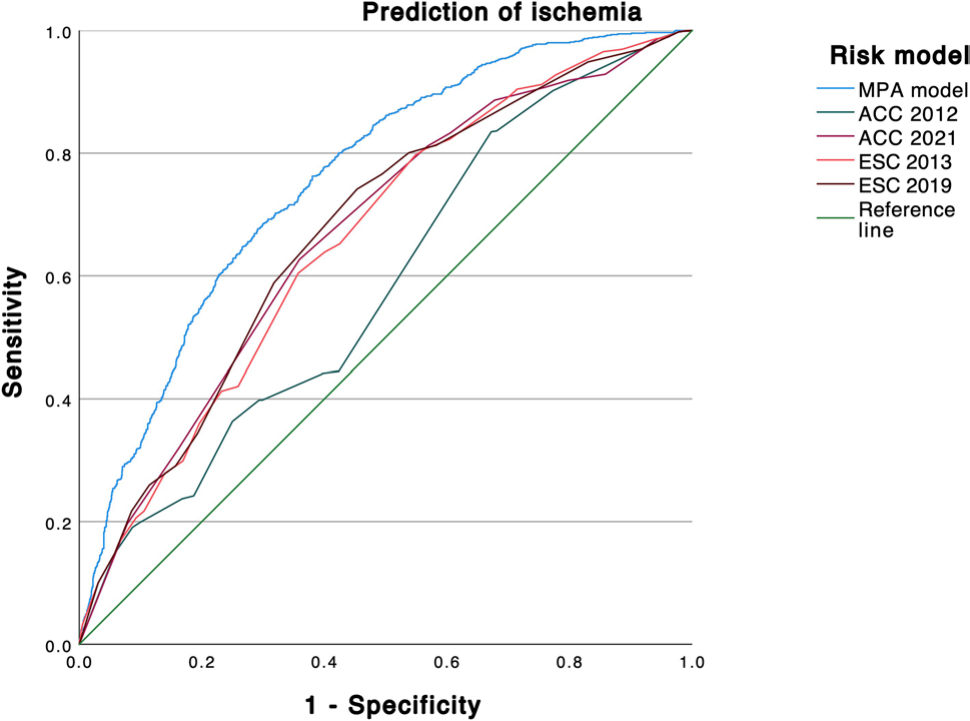
ROC curve of different models to predict ischemia on PET. The figure shows the receiver operating characteristic (ROC) curves of 5 different scores to predict ischemia on PET. The MPA model has a statistically significant higher AUC (*p* < 0.0001)

The AUC of the MPA for ischemia was 0.758 (95% CI 0.739–0.777), and significantly higher than the AUC of every other score tested (*p* < 0.0001 each). The overall ROC curve and AUC values are depicted in Fig. 1 and Table 3. The ESC 2019 and ACC 2021 scores performed second and third best with an AUC of 0.673 and 0.667, respectively.

**Table 3:** Comparison of test performance

Model	AUC	LL 95% CI	UL 95% CI
MPA model	0.758	0.739	0.777
ACC 2012	0.585	0.562	0.608
ACC 2021	0.667	0.645	0.689
ESC 2013	0.661	0.639	0.683
ESC 2019	0.673	0.651	0.695

The table indicates the area under the curve (AUC) of different pre-test probability scores for the prediction of ischemia. *CI* confidence interval, *LL* lower limit, *UL* upper limit

### Distribution of patients with ischemia according to PTP categories

**Fig. 2:**
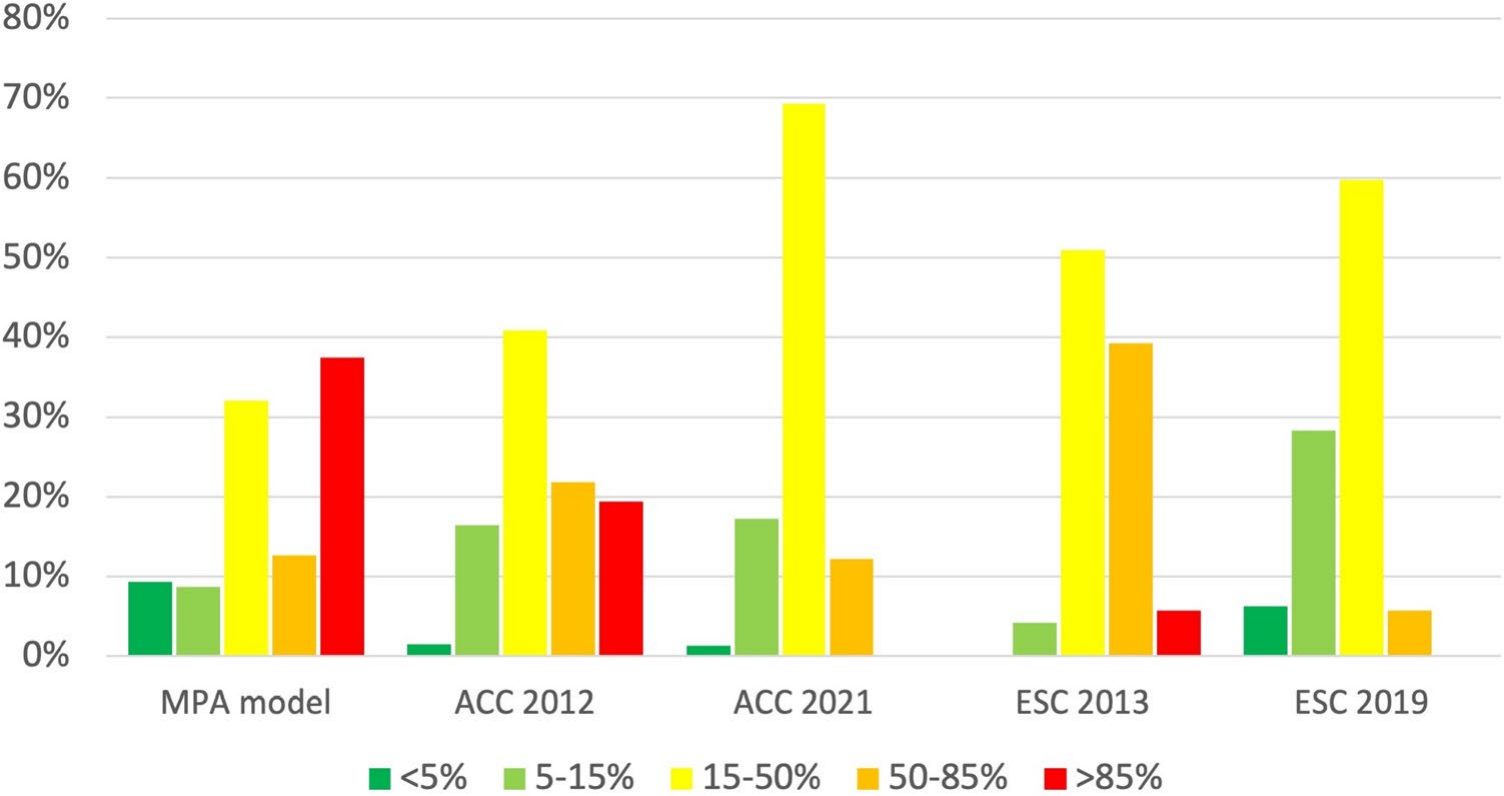
Distribution of patients across pre-test probability categories depending on the risk score used. The bar chart indicates the proportion of patients within the corresponding pre-test probability (PTP) category. The MPA model stratifies more evenly across all five PTP categories

Not all scores allocated patients to all available five PTP categories, e.g. no very high PTP category in ACC 2021/ESC 2019 and no very low PTP category in ESC 2013. Certain categories entailed only a small proportion of the cohort (e.g. 1.2% in the very low PTP category with ACC 2021 score). Comparing the relative distribution of patients, the MPA model stratified patients more evenly over the five PTP categories as visually illustrated in Fig. 2. The minimal and maximal proportion per category was 8.6% and 37.4%, compared to 0.0% and 69.3% in other scores. The proportion of patients in the 15–85% range was considerably lower with the MPA model (MPA 44.6%, ACC 2012 62.7%, ESC 2019 65.5%, ACC 2021 81.5%, ESC 2013 90.2%, *p* < 0.001 each) as summarised in Table 4. Only in the high and very high PTP category, the algorithm overestimated the true prevalence of ischemia. This finding was similar for all other scores.

**Table 4:**
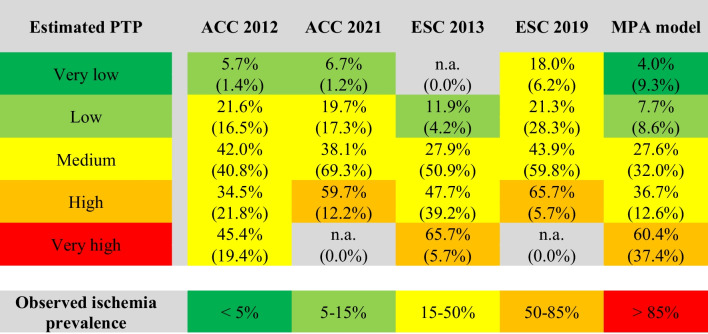
Comparison of the MPA model with four common pre-test probability scores

Table indicating the distribution of patients within their predicted pre-test probability (PTP) category according to four common PTP scores and the MPA model. The percentage at the top of the table cell indicates the observed prevalence of ischemia within each category and is colour-coded. The percentage at the bottom of the cell in parentheses represent the percentage of patients in the corresponding category

In the very low PTP category, the MPA model was the only tool, which correctly estimated ischemia prevalence to be < 5%. Not only did the ACC 2012 and ACC 2021 scores slightly underestimate prevalence (5–7%), they also only allocated 1.2–1.4% of patients in this PTP category compared to 9.3% by the MPA algorithm. The ESC 2013 score was not able to allocate patients to the very low PTP category. The ESC 2019 score significantly underestimated true prevalence of ischemia (18% in the < 5% PTP category).

Apart from the MPA model, only the ESC 2013 score predicted ischemia correctly in the low PTP category. But, it allocated less than half of patients in this category than the MPA model (4.2% vs. 8.6%, respectively, *p* < 0.001).

Combining the first two PTP categories (< 15%), only the MPA model and ESC 2013 score predicted ischemia correctly (5.8% and 11.9%, respectively), but the MPA model was able to allocate > 4 times more patients (17.9% vs. 4.2%, *p* < 0.001) correctly.

### Test characteristics to exclude ischemia on PET

Test characteristics of the MPA model and the two ACC and ESC scores to exclude ischemia on PET are summarised in Table 5. Using the threshold < 15% PTP, the MPA model showed an excellent test performance with a sensitivity of 97.3%, a NLR of 0.099 and a DOR of 13.390. Using the threshold < 5%, the MPA’s sensitivity, NLR and DOR were 99.1%, 0.063 and 18.400, respectively.

**Table 5:** Test characteristics of the MPA model compared to four common pre-test probability scores

	Sensitivity	Specificity	NPV	PPV	PLR	NLR	DOR	FNR	FPR	*n* =	% patients
Low PTP (< 15%)											
MPA model	97.3%	26.9%	94.5%	44.0%	1.332	0.099	13.390	2.7%	73.1%	433	17.9%
ACC 2012	90.2%	22.7%	79.7%	40.8%	1.167	0.432	2.699	9.8%	77.3%	433	17.9%
ACC 2021	90.7%	23.9%	81.4%	41.3%	1.193	0.386	3.088	9.3%	76.1%	447	18.5%
ESC 2013	98.7%	5.9%	88.1%	38.2%	1.048	0.228	4.587	1.3%	94.1%	101	4.2%
ESC 2019	80.8%	43.6%	79.4%	45.8%	1.432	0.440	3.252	19.2%	56.4%	834	34.5%
Very low PTP (< 5%)											
MPA model	99.1%	14.2%	96.4%	40.5%	1.155	0.063	18.400	0.9%	85.8%	224	9.3%
ACC 2012	99.8%	2.2%	94.3%	37.6%	1.020	0.103	9.931	0.2%	97.8%	35	1.4%
ACC 2021	99.8%	1.8%	93.3%	37.5%	1.016	0.121	8.398	0.2%	98.2%	30	1.2%
ESC 2013	NA	NA	NA	NA	NA	NA	NA	NA	NA	0	0.0%
ESC 2019	97.0%	8.1%	82.0%	38.4%	1.055	0.372	2.837	3.0%	91.9%	150	6.2%
Very high PTP (> 85%)											
MPA model	61.0%	76.4%	76.9%	60.4%	2.589	0.510	5.073	39.0%	23.6%	905	37.4%
ACC 2012	23.7%	83.2%	64.9%	45.4%	1.410	0.917	1.538	76.3%	16.8%	469	19.4%
ACC 2021	NA	NA	NA	NA	NA	NA	NA	NA	NA	0	0.0%
ESC 2013	10.0%	96.9%	64.6%	65.7%	3.245	0.928	3.495	90.0%	3.1%	137	5.7%
ESC 2019	NA	NA	NA	NA	NA	NA	NA	NA	NA	0	0.0%

The table indicates test characteristics of the MPA model and four commonly used pre-test probability scores. Three different cut-offs were defined (low: < 15% PTP; very low: < 5% PTP; very high: > 85% PTP). *DOR* diagnostic odds ratio, *FNR* false negative rate, *FPR* false positive rate, *NLR* negative likelihood ratio, *NPV* negative predictive value, *PLR* positive likelihood ratio, *PPV* positive predictive value, *PTP* pre-test probability

The MPA was the only score with a NLR below 0.1 which is regarded a good test for exclusion of a disease [24]. Furthermore, DOR was highest compared to the other scores suggesting best diagnostic accuracy.

### Test characteristics to detect ischemia on PET

Only 3 scores predicted patients to have very high PTP (> 85%) as shown in Table 5. None of the models had a PLR above 10, which would be necessary to be a good rule-in test. Overall, the MPA model had the highest diagnostic accuracy, but the ESC 2013 had a higher PLR and PPV. However, the ESC 2013 allocated significantly less patient in the very high PTP category (5.7% vs. 37.4%, *p* < 0.001). The ACC 2021 and ESC 2019 score did not provide PTP values above 52%.

### Test characteristics in different subgroups

In the subgroup analysis, the MPA model performed not as good as in the whole patient cohort, but still better compared to the other risk scores as shown in Table S1.

The test characteristics of the analysed scores in different subgroups (with/without CAD, female/male patients) are depicted in Tables S2-4.

The MPA discriminated better in patients with no CAD compared to prior CAD (higher AUC and DOR, lower NLR). Still, test characteristics of the MPA model were better compared to the other four scores. AUC was higher in female patients compared to male patients (0.770 vs. 0.708). Using the thresholds of < 15% and < 5%, the MPA model had the highest DOR and lowest NLR in all subgroups tested.

In all subgroups, diagnostic accuracy was best with the MPA, except for patients without CAD in whom DOR was higher with ESC 2013 when it comes to patients with very high PTP (however, less patients were allocated than with MPA).

### Correlation of MPA model score with ischemia

**Fig. 3:**
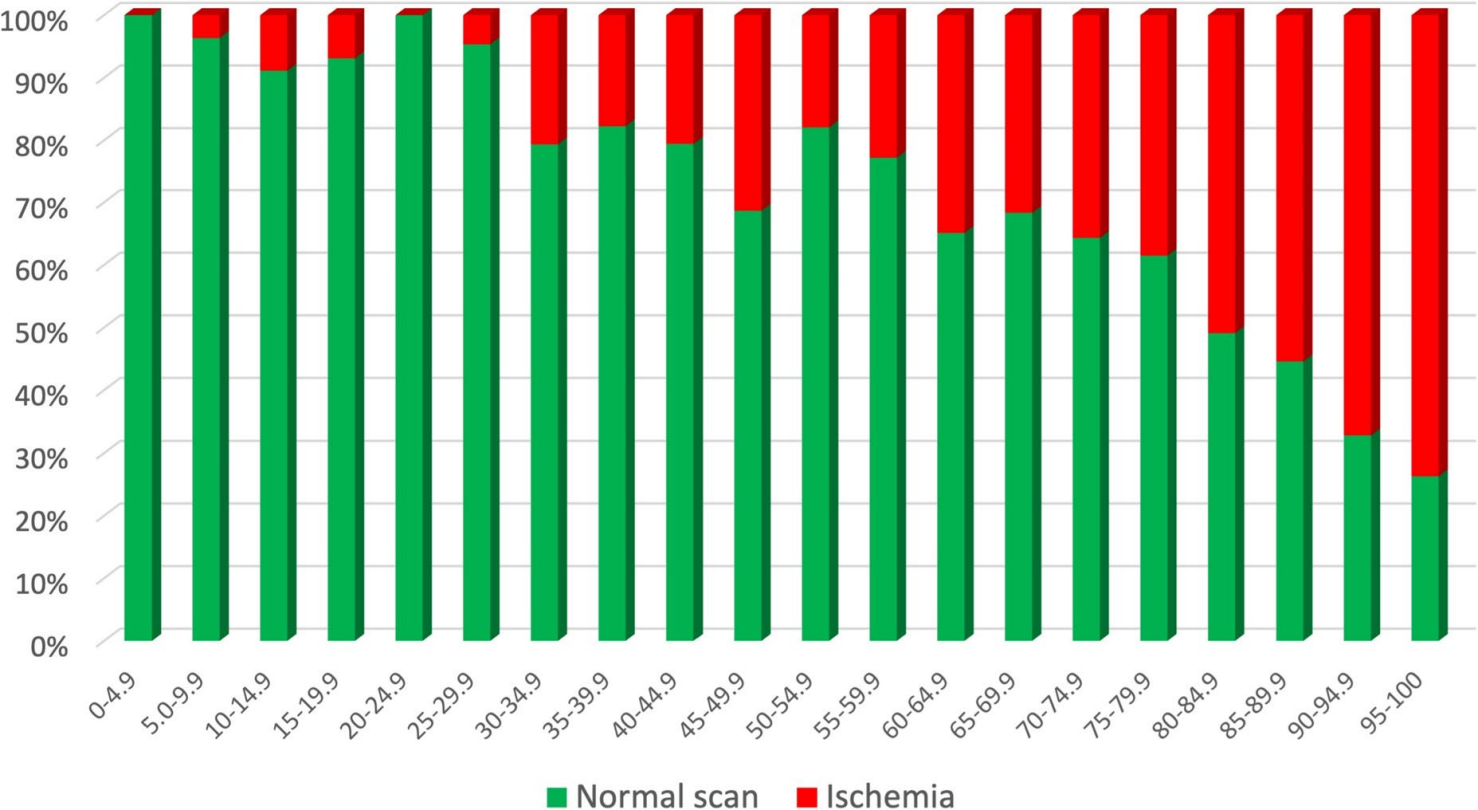
Prevalence of ischemia stratified by MPA model. The figure indicates the relative proportion if ischemia in PET depending on the value derived from the MPA model. The higher the MPA value, the more likely a patient has ischemia

With higher MPA model values, the prevalence of ischemia increases from 0% in the lowest group to 73.7% in the highest, which is visualised in Fig. 3.

## Discussion

The main findings of this study are as follows: (1) The MPA model provided more accurate prediction of ischemia than the recommended PTP models (ESC 2013, ESC 2019, ACC 2012, ACC 2021). (2) The MPA model was the only model which correctly identified patients with a very low likelihood of ischemia. (3) The MPA model improved stratification across the whole PTP spectrum and reduced the proportion of patients in the intermediate range of 15–85% PTP by 28.9% (ACC 2012)–50.6% (ESC 2019). (4) The MPA model worked in patients without and with prior CAD, although it performed better in patients without prior CAD. Therefore, it should probably be used predominantly in patient cohorts without prior CAD. Hence, the MPA model is a useful tool to improve individualised assessment of pre-test probability and preselect patients for advanced cardiac testing. Furthermore, it could prevent patients with low probability of ischemia from unnecessary downstream tests, radiation exposure and costs. Therefore, it is a clear advancement in the direction of PPPM.

### Current PTP tools are insufficient for patient preselection

Despite their easy use, the traditional risk prediction tools have two significant limitations. First, they either classify a substantial number of patients to have PTP < 15% with an insufficient sensitivity (80.8–90.7%) only (hence significantly underestimate the true prevalence), or they have an excellent sensitivity, but allocate a small proportion of patients in this PTP category only. Second, they allocate the majority of patients in the 15–85% range in which non-invasive imaging is recommended. Consequently, they are not useful in reducing the number of unnecessary non-invasive testing.

### Comparison to earlier studies with the MPA model

The MPA model may overcome these issues to a clinically relevant extent with a more even distribution across PTP categories while maintaining an excellent sensitivity, NPV, NLR and FPR.

The MPA model’s overall AUC of 0.758 was good [24] and it performed clearly better compared to all other scores, also in the subgroup analyses. Still, the overall AUC was lower than reported in the earlier studies (original validation cohort Basel MPA 0.824 [20], LURIC validation 0.87 [17], Eurlings 0.87 [16]). This is most likely because the algorithm was trained and validated in previous works to detect the anatomic presence of CAD documented by invasive coronary angiography but not ischemia. In the present study, detection of ischemia by PET was used. A coronary vessel with an anatomic stenosis of > 50% as defined in the previous studies [20] does not necessarily translate into ischemia. In a sub-study of the COURAGE trial, Shaw et al. showed that approximately 40% of patients with at least one ≥ 70% stenosis had no or minimal ischemia only [25]. In the FAME trial, coronary stenoses in the range of 50–70% and 71–90% were not functionally significant in 65% and 20%, respectively [26]. Hence, this fact may explain at least in part the lower discriminatory power in the current study using the endpoint of ischemia, if compared directly to the initial MPA studies. Similar findings apply for the ACC and ESC scores [27, 28].

### Performance of MPA model in subgroups

Despite the model being developed and trained in a cohort of patients without prior CAD, the MPA algorithm also performed acceptable in the subgroups (e.g. prior CAD). The AUC of each subgroup was lower than the AUC of the overall model, except for female patients where it was even slightly higher. The fact that both groups (with/without prior CAD) had worse AUC than the overall population is most likely because factors attributing for “prior CAD” significantly contribute to the model to estimate prevalence of CAD. The better AUC in female patients is probably because female patients had a lower prevalence of prior CAD.

Overall, the AUC of the MPA model was higher than all the PTP scores in each subgroup, highlighting the better discriminatory power and consistency of the test. Additionally, a higher MPA model score correlated well with the prevalence of ischemia. This may confirm the validity of this model also on a pathophysiological basis.

### Potential field of application

A big advantage of the MPA algorithm is its ability to discriminate patients better across the whole spectrum of PTP, especially in the low- and very-low-risk categories. It exceeded the other models to correctly identify patients who have a very low prevalence of ischemia. If a certain cut-off for post-test probability was clinically accepted to abstain from testing (e.g. 5% or 10%, as proposed by certain authors [13]), this algorithm could be used to omit non-invasive testing in a significant number of patients.

The test characteristics to allocate patients in the very high-risk category (> 85%) were not as good as on the other side of the spectrum. This was most likely due to over-estimation of actual prevalence of ischemia, which was also observed with the other scores [27, 28]. This is most likely because all of them were developed and calibrated in cohorts where coronary artery disease was defined by luminal stenosis from an anatomical test (invasive angiography or computed tomography coronary angiography (CTCA)). As described above, significant luminal narrowing does not necessarily translate into ischemia. The clinical relevance of this slight overestimation of the prevalence of CAD appears insignificant since all of these high-risk patients need an advanced testing strategy anyway, be it non-invasive functional testing or an invasive angiogram. Hence, the MPA model is a better “rule-out” than “rule-in” test. Still, with the MPA’s false positive rate of 23.6%, this proportion is clearly below the prevalence of non-obstructed coronary arteries on routine angiograms as reported in certain cohorts (62.4%)29.

### Comparison of study findings to published works

Miller et al. described a similar approach in a large multi-centre, international registry with > 20,000 patients [19]. They used patient specific data available prior to the scan and a machine learning–based algorithm to predict an abnormal myocardial perfusion [19]. The AUC to predict an abnormal scan was 0.762 (95% CI 0.750–0.774), which was similar to our MPA algorithm (0.758, 95% CI 0.739–0.777). Using their ultra-high sensitive threshold (which is approximately equivalent to our low PTP threshold (PTP < 15%)), test characteristics were comparable (sensitivity: 96% vs. 97%; NPV: 95% vs. 95%; 15.5% vs. 17.9% of patients below threshold). But, our very low PTP threshold exceeded the described ultra-sensitive threshold with a sensitivity of 99% and NPV of 96%. However, comparability is limited because Miller et al. included two variables in their model (prior CAD and past myocardial infarction) which account for the major part of the model. Even without including these two important factors, our model outperformed the described model if the < 5% cut-off is used. Furthermore, they did not include biomarkers and the endpoints differed significantly (SDS ≥ 2 on PET (this publication) vs. SSS ≥ 3 on SPECT (Miller)).

In another study, Ismaeel and colleagues compared an artificial neural network (ANN) with two older PTP tools (Diamond Forrester, Morise) to predict ischemia [18]. Similar to our study, the AI model outperformed the PTP tools and had a better discriminator power and good test characteristics to rule out ischemia (sensitivity 91%, negative predictive value 98%). The AUC of the ANN model was slightly lower than our MPA model (0.7 vs. 0.76). Comparability with this small study (*n* = 486) is difficult, because they used PTP tools which are not recommended anymore in the guidelines, the endpoint ischemia was not well defined and two different functional tests (SPECT, stress echocardiography) were used, which are less sensitive and less specific than PET.

### Using the MPA model

The test characteristics of a given model strongly depend on the prevalence of the disease. Hence, cut-off points need to be adjusted depending on the cohort being tested. Therefore, two different calibrations of the MPA model are available [16, 17, 20] (MPA model and MPA model low risk; Table S5). Since these were calibrated in different cohorts with different prevalence of CAD, they use different cut-off points and must not be swapped interchangeably. As shown in Table S6, the MPA low-risk model [16] stratifies better in the high and very high-risk categories, but significantly underestimates ischemia prevalence in the low-risk category of the current study cohort.

Therefore, in order to ensure accurate risk stratification using the MPA model, it is important to select the appropriate cut-off points depending on the clinical setting of the patient (e.g. as used for risk stratification or screening in a general practitioner’s (GP) office vs. a diagnostic test in patients referred to a cardiologist’s office or hospital).

Despite better ischemia prediction in the very high PTP category (> 85%) compared to the other scores, the MPA model performed best to exclude ischemia. It could be used as a gatekeeper to reduce costs while maintaining its excellent test characteristics (cut-off > 15% PTP, sensitivity 97.3%, NPV 94.5%, NLR 0.099). Based on clinical information, biomarkers and ECG findings, it could be applied by primary care physicians to triage patients before they are referred for further downstream cardiac testing.

### Limitations

Data from this project arise from a single centre. Images were analysed according to current guidelines by a small, steady and experienced team of Cardiologists and Nuclear Medicine Specialists reaching consensus. Hence, data interpretation was performed in a standardised and homogeneous way.

The four scores (ESC, ACC) were initially developed to assess pre-test probability of significant luminal stenosis in patients without prior CAD. We applied these scores in a mixed population with and without prior CAD which could limit the scores’ overall performance. However, we provide the subgroup analysis for both patient with and patients without prior CAD.

## Conclusion and outlook

The memetic pattern–based algorithm model outperformed traditional tools in the prediction of ischemia. It was the only tool which correctly estimated prevalence of ischemia in the very low PTP category (< 5% PTP), and it excluded ischemia with an excellent sensitivity and negative predictive value. Furthermore, it allocated patients more evenly across all PTP categories and reduced the proportion of patients in the intermediate range (PTP 15–85%) by 29 to 51%:

1. Predictive approach: The MPA’s very high sensitivity (> 99%) to detect ischemia and the ability to identify patients with a very low prevalence of ischemia has several important clinical implications. Patients at risk or with symptoms suspicious for CAD usually present at the GP’s office. The clinical assessment is often completed by ECG and laboratory workup. Subsequently, patients are referred to cardiologists and/or further downstream testing. With the described algorithm, “very low risk” patients could be easily and safely identified, and ischemia be excluded during the first GP visit using the already available data. Patients with elevated risk could be identified and sent for further testing. This predictive approach could provide a safe and reliable exclusion test to the GP and a precise, comprehensive assessment to the individual patients.
2. Targeted prevention: Patients at risk according to the MPA model should be further evaluated and be referred for advanced cardiac testing. Moreover, the MPA model could prevent patients without ischemia from unnecessary radiation and stress agent exposure.
3. Personalisation of medical services: Using the MPA model, the diagnostic pathway could be tailored to the individual patient. On one hand, this would include deferring patients without significant disease from cardiac tests. On the other hand, it would ensure that patients who benefit from advanced testing, will be tested.

In addition, slots for functional test are limited. With the expected demographic changes, the demand for such tests is expected to rise. Instead of increasing testing capacities of these expensive tests (e.g. PET scan ~ 3000 Swiss Francs), the MPA model could be implemented as gatekeeper. This could reduce the number of normal scans and reduce healthcare costs on a population level.

In summary, the MPA model offers a step towards a more predictive, preventive and personalised medicine (PPPM).

### Supplementary Information

Below is the link to the electronic supplementary material.

Supplementary file1 (DOCX 112 KB)

Supplementary file2 (DOCX 17.9 KB)

## Data Availability

Data available upon reasonable request.

## Abbreviations

ANN: Artificial neural network
AUC: Area under the curve
BMI: Body mass index
CAC: Coronary artery calcification
CAD: Coronary artery disease
CACS: Coronary artery calcium score
CTCA: Computed tomography coronary angiography
DF: Diamond-Forrester
DOR: Diagnostic odds ratio
FNR: False negative rate
FPR: False positive rate
GP: General practitioner
MPA: Memetic pattern–based algorithm
NLR: Negative likelihood ratio
NPV: Negative predictive value
PET: Positron emission tomography
PLR: Positive likelihood ratio
PPPM: Predictive, preventive and personalised medicine
PPV: Positive predictive value
PTP: Pre-test probability
ROC: Receiver operating characteristic
SD: Standard deviation

## Code availability

The code is of MPA model is not available due to proprietary reasons.
