# Supplementary material for: Artificial intelligence to improve ischemia prediction in Rubidium Positron Emission Tomography—a validation study
Source: EPMA J. 2023 Nov 15;14(4):631–43. doi: 10.1007/s13167-023-00341-5 (PMC10713509; doi:10.1007/s13167-023-00341-5)
Supplement: Supplementary file 1 — Supplementary file1 (DOCX 112 KB) [file 13167_2023_341_MOESM1_ESM.docx]

# Supplemental materials

## PET image reconstruction details:

For reconstruction, an ordered subset expectation maximization algorithm (OSEM, matrix 512 x 512, 3 iterations, 21 subsets, gauss-filtering, 8 mm full-width half-maximum) was used for static and dynamic images. Random, scatter, attenuation and decay corrections were automatically applied to the emission data. Automatic motion correction was enabled. The following framesets were used for reconstruction of dynamic images: 1s delay, 12x10 s, 4x30 s, 1x60 s, 1x120s. The last four minutes of acquisition were used for reconstruction of all images (static and ECG-gated).

Supplement Table S1: Comparison of test performance in different subgroups

| **Risk model** | **AUC** | **LL 95% CI** | **UL 95% CI** | |
| --- | --- | --- | --- | --- |
| **no CAD (n = 1297)** | | | |  |
| MPA model | 0.725 | 0.693 | 0.757 |  |
| ACC 2012 | 0.599 | 0.561 | 0.637 | |
| ACC 2021 | 0.667 | 0.630 | 0.703 | |
| ESC 2013 | 0.678 | 0.642 | 0.713 | |
| ESC 2019 | 0.686 | 0.650 | 0.722 | |
| **known CAD (n = 1120)** | | | |  |
| MPA model | 0.652 | 0.619 | 0.684 |  |
| ACC 2012 | 0.555 | 0.521 | 0.589 | |
| ACC 2021 | 0.603 | 0.569 | 0.636 | |
| ESC 2013 | 0.599 | 0.566 | 0.633 | |
| ESC 2019 | 0.610 | 0.576 | 0.643 | |
| **male patients (n = 1653)** | | | |  |
| MPA model | 0.708 | 0.683 | 0.733 |  |
| ACC 2012 | 0.573 | 0.546 | 0.601 | |
| ACC 2021 | 0.597 | 0.570 | 0.625 | |
| ESC 2013 | 0.613 | 0.586 | 0.640 | |
| ESC 2019 | 0.622 | 0.595 | 0.649 | |
| **female patients (n = 764)** | | | |  |
| MPA model | 0.770 | 0.729 | 0.810 |  |
| ACC 2012 | 0.528 | 0.476 | 0.581 | |
| ACC 2021 | 0.539 | 0.484 | 0.593 | |
| ESC 2013 | 0.584 | 0.533 | 0.635 | |
| ESC 2019 | 0.585 | 0.534 | 0.637 | |

The table indicates the area under the curve (AUC) of different pre-test probability scores for the prediction of ischemia in different subgroups. CI: confidence interval. LL: lower limit. UL: upper limit: SE: standard error.

Supplemental Table S2: Test characteristics of the MPA model compared to four common pre-test probability scores in different subgroups (cut-off: low risk <15%)

|  | **Sensitivity** | **Specificity** | **NPV** | **PPV** | **PLR** | **NLR** | **DOR** | **FNR** | **FPR** | **n =** | **% patients** |
| --- | --- | --- | --- | --- | --- | --- | --- | --- | --- | --- | --- |
| **all patients (n = 2417)** | | | | | | | | | | | |
| MPA model | 97.3% | 26.9% | 94.5% | 44.0% | 1.332 | 0.099 | 13.390 | 2.7% | 73.1% | 433 | 17.9% |
| ACC 2012 | 90.2% | 22.7% | 79.7% | 40.8% | 1.167 | 0.432 | 2.699 | 9.8% | 77.3% | 433 | 17.9% |
| ACC 2021 | 90.7% | 23.9% | 81.4% | 41.3% | 1.193 | 0.386 | 3.088 | 9.3% | 76.1% | 447 | 18.5% |
| ESC 2013 | 98.7% | 5.9% | 88.1% | 38.2% | 1.048 | 0.228 | 4.587 | 1.3% | 94.1% | 101 | 4.2% |
| ESC 2019 | 80.8% | 43.6% | 79.4% | 45.8% | 1.432 | 0.440 | 3.252 | 19.2% | 56.4% | 834 | 34.5% |
| **without CAD (n = 1297)** | | | | | | | | | | | |
| MPA model | 93.3% | 36.6% | 95.7% | 26.2% | 1.470 | 0.185 | 7.965 | 6.7% | 63.4% | 399 | 30.8% |
| ACC 2012 | 86.9% | 26.7% | 89.4% | 22.2% | 1.186 | 0.490 | 2.417 | 13.1% | 73.3% | 312 | 24.1% |
| ACC 2021 | 87.7% | 28.9% | 90.7% | 22.9% | 1.233 | 0.426 | 2.898 | 12.3% | 71.1% | 333 | 25.7% |
| ESC 2013 | 98.4% | 7.7% | 95.2% | 20.4% | 1.066 | 0.207 | 5.140 | 1.6% | 92.3% | 84 | 6.5% |
| ESC 2019 | 79.8% | 49.3% | 91.0% | 27.5% | 1.573 | 0.411 | 3.830 | 20.2% | 50.7% | 566 | 43.6% |
| **with CAD (n = 1120)** | | | | | | | | | | | |
| MPA model | 98.9% | 5.7% | 79.4% | 58.7% | 1.049 | 0.191 | 5.493 | 1.1% | 94.3% | 34 | 3.0% |
| ACC 2012 | 91.5% | 13.9% | 54.5% | 59.1% | 1.062 | 0.614 | 1.731 | 8.5% | 86.1% | 121 | 10.8% |
| ACC 2021 | 91.9% | 13.1% | 54.4% | 58.9% | 1.057 | 0.618 | 1.712 | 8.1% | 86.9% | 114 | 10.2% |
| ESC 2013 | 98.8% | 1.9% | 52.9% | 57.8% | 1.007 | 0.655 | 1.538 | 1.2% | 98.1% | 17 | 1.5% |
| ESC 2019 | 81.2% | 30.9% | 54.9% | 61.5% | 1.176 | 0.606 | 1.941 | 18.8% | 69.1% | 268 | 23.9% |
| **male (n = 1653)** | | | | | | | | | | | |
| MPA model | 98.8% | 10.9% | 91.6% | 48.1% | 1.109 | 0.110 | 10.070 | 1.2% | 89.1% | 107 | 6.5% |
| ACC 2012 | 98.0% | 4.9% | 74.6% | 46.2% | 1.030 | 0.408 | 2.523 | 2.0% | 95.1% | 59 | 3.6% |
| ACC 2021 | 98.3% | 3.7% | 71.7% | 46.0% | 1.020 | 0.472 | 2.161 | 1.7% | 96.3% | 46 | 2.8% |
| ESC 2013 | NA | NA | NA | NA | NA | NA | NA | NA | NA | 0 | 0.0% |
| ESC 2019 | 88.8% | 23.4% | 71.5% | 49.2% | 1.160 | 0.477 | 2.432 | 11.2% | 76.6% | 295 | 17.8% |
| **female (n = 764)** | | | | | | | | | | | |
| MPA model | 89.7% | 50.2% | 95.4% | 29.7% | 1.802 | 0.206 | 8.751 | 10.3% | 49.8% | 326 | 42.7% |
| ACC 2012 | 49.7% | 48.6% | 80.5% | 18.5% | 0.967 | 1.035 | 0.934 | 50.3% | 51.4% | 374 | 49.0% |
| ACC 2021 | 51.7% | 53.5% | 82.5% | 20.7% | 1.112 | 0.903 | 1.231 | 48.3% | 46.5% | 401 | 52.5% |
| ESC 2013 | 91.7% | 14.4% | 88.1% | 20.1% | 1.071 | 0.576 | 1.861 | 8.3% | 85.6% | 101 | 13.2% |
| ESC 2019 | 39.3% | 72.9% | 83.7% | 25.3% | 1.448 | 0.833 | 1.739 | 60.7% | 27.1% | 539 | 70.5% |

Table indicates test characteristics of the MPA model and four commonly used pre-test probability scores. Cut-off was defined as low PTP (<15%). CAD: coronary artery disease. DOR: diagnostic odds ratio. FNR: false negative rate. FPR: false positive rate. NLR: negative likelihood ratio. NPV: negative predictive value. PLR: positive likelihood ratio. PPV: positive predictive value. PTP: pre-test probability.

Supplemental Table S3: Test characteristics of the MPA model compared to four common pre-test probability scores in different subgroups (cut-off: very low risk <5%)

|  | **Sensitivity** | **Specificity** | **NPV** | **PPV** | **PLR** | **NLR** | **DOR** | **FNR** | **FPR** | **n =** | **% patients** |
| --- | --- | --- | --- | --- | --- | --- | --- | --- | --- | --- | --- |
| **all patients (n = 2417)** | | | | | | | | | | | |
| MPA model | 99.1% | 14.2% | 96.4% | 40.5% | 1.155 | 0.063 | 18.400 | 0.9% | 85.8% | 224 | 9.3% |
| ACC 2012 | 99.8% | 2.2% | 94.3% | 37.6% | 1.020 | 0.103 | 9.931 | 0.2% | 97.8% | 35 | 1.4% |
| ACC 2021 | 99.8% | 1.8% | 93.3% | 37.5% | 1.016 | 0.121 | 8.398 | 0.2% | 98.2% | 30 | 1.2% |
| ESC 2013 | NA | NA | NA | NA | NA | NA | NA | NA | NA | 0 | 0.0% |
| ESC 2019 | 97.0% | 8.1% | 82.0% | 38.4% | 1.055 | 0.372 | 2.837 | 3.0% | 91.9% | 150 | 6.2% |
| **without CAD (n = 1297)** | | | | | | | | | | | |
| MPA model | 98.0% | 19.7% | 97.6% | 22.7% | 1.221 | 0.101 | 12.120 | 2.0% | 80.3% | 211 | 16.3% |
| ACC 2012 | 99.6% | 3.0% | 96.9% | 19.8% | 1.026 | 0.134 | 7.674 | 0.4% | 97.0% | 32 | 2.5% |
| ACC 2021 | 99.6% | 2.7% | 96.6% | 19.8% | 1.023 | 0.148 | 6.911 | 0.4% | 97.3% | 29 | 2.2% |
| ESC 2013 | NA | NA | NA | NA | NA | NA | NA | NA | NA | 0 | 0.0% |
| ESC 2019 | 97.6% | 9.8% | 94.4% | 20.7% | 1.082 | 0.244 | 4.435 | 2.4% | 90.2% | 108 | 8.3% |
| **with CAD (n = 1120)** | | | | | | | | | | | |
| MPA model | 99.5% | 2.1% | 76.9% | 58.0% | 1.017 | 0.221 | 4.602 | 0.5% | 97.9% | 13 | 1.2% |
| ACC 2012 | 99.8% | 0.4% | 66.7% | 57.7% | 1.003 | 0.368 | 2.723 | 0.2% | 99.6% | 3 | 0.3% |
| ACC 2021 | 99.8% | 0.0% | 0.0% | 57.6% | 0.998 | NA | NA | 0.2% | 100.0% | 1 | 0.1% |
| ESC 2013 | NA | NA | NA | NA | NA | NA | NA | NA | NA | 0 | 0.0% |
| ESC 2019 | 96.7% | 4.4% | 50.0% | 57.9% | 1.012 | 0.736 | 1.374 | 3.3% | 95.6% | 42 | 3.8% |
| **male (n = 1653)** | | | | | | | | | | | |
| MPA model | 99.7% | 5.2% | 95.9% | 46.8% | 1.052 | 0.051 | 20.630 | 0.3% | 94.8% | 49 | 3.0% |
| ACC 2012 | 99.9% | 1.0% | 90.0% | 45.7% | 1.009 | 0.133 | 7.577 | 0.1% | 99.0% | 10 | 0.6% |
| ACC 2021 | 99.7% | 1.2% | 84.6% | 45.7% | 1.010 | 0.218 | 4.635 | 0.3% | 98.8% | 13 | 0.8% |
| ESC 2013 | NA | NA | NA | NA | NA | NA | NA | NA | NA | 0 | 0.0% |
| ESC 2019 | 97.9% | 5.1% | 74.2% | 46.3% | 1.031 | 0.417 | 2.475 | 2.1% | 94.9% | 62 | 3.8% |
| **female (n = 764)** | | | | | | | | | | | |
| MPA model | 95.9% | 27.3% | 96.6% | 23.6% | 1.319 | 0.152 | 8.700 | 4.1% | 72.7% | 175 | 22.9% |
| ACC 2012 | 99.3% | 3.9% | 96.0% | 19.5% | 1.033 | 0.178 | 5.808 | 0.7% | 96.1% | 25 | 3.3% |
| ACC 2021 | 100.0% | 2.7% | 100.0% | 19.4% | 1.028 | NA | NA | 0.0% | 97.3% | 17 | 2.2% |
| ESC 2013 | NA | NA | NA | NA | NA | NA | NA | NA | NA | 0 | 0.0% |
| ESC 2019 | 92.4% | 12.4% | 87.5% | 19.8% | 1.055 | 0.610 | 1.731 | 7.6% | 87.6% | 88 | 11.5% |

Table indicates test characteristics of the MPA model and four commonly used pre-test probability scores. Cut-off was defined as very low PTP (<5%). CAD: coronary artery disease. DOR: diagnostic odds ratio. FNR: false negative rate. FPR: false positive rate. NLR: negative likelihood ratio. NPV: negative predictive value. PLR: positive likelihood ratio. PPV: positive predictive value. PTP: pre-test probability.

Supplemental Table S4: Test characteristics of the MPA model compared to four common pre-test probability scores in different subgroups (cut-off: very high risk >85%)

|  | **Sensitivity** | **Specificity** | **NPV** | **PPV** | **PLR** | **NLR** | **DOR** | **FNR** | **FPR** | **n =** | **% patients** |
| --- | --- | --- | --- | --- | --- | --- | --- | --- | --- | --- | --- |
| **all patients (n = 2417)** | | | | | | | | | | | |
| MPA model | 61.0% | 76.4% | 76.9% | 60.4% | 2.589 | 0.510 | 5.073 | 39.0% | 23.6% | 905 | 37.4% |
| ACC 2012 | 23.7% | 83.2% | 64.9% | 45.4% | 1.410 | 0.917 | 1.538 | 76.3% | 16.8% | 469 | 19.4% |
| ACC 2021 | NA | NA | NA | NA | NA | NA | NA | NA | NA | 0 | 0.0% |
| ESC 2013 | 10.0% | 96.9% | 64.6% | 65.7% | 3.245 | 0.928 | 3.495 | 90.0% | 3.1% | 137 | 5.7% |
| ESC 2019 | NA | NA | NA | NA | NA | NA | NA | NA | NA | 0 | 0.0% |
| **without CAD (n = 1297)** | | | | | | | | | | | |
| MPA model | 32.9% | 87.9% | 84.5% | 39.7% | 2.732 | 0.763 | 3.582 | 67.1% | 12.1% | 209 | 16.1% |
| ACC 2012 | 26.6% | 83.6% | 82.5% | 28.2% | 1.625 | 0.878 | 1.851 | 73.4% | 16.4% | 238 | 18.4% |
| ACC 2021 | NA | NA | NA | NA | NA | NA | NA | NA | NA | 0 | 0.0% |
| ESC 2013 | 7.9% | 98.0% | 81.5% | 48.8% | 3.949 | 0.940 | 4.204 | 92.1% | 2.0% | 41 | 3.2% |
| ESC 2019 | NA | NA | NA | NA | NA | NA | NA | NA | NA | 0 | 0.0% |
| **with CAD (n = 1120)** | | | | | | | | | | | |
| MPA model | 71.9% | 51.2% | 57.3% | 66.7% | 1.473 | 0.549 | 2.685 | 28.1% | 48.8% | 696 | 62.1% |
| ACC 2012 | 22.6% | 82.1% | 43.9% | 63.2% | 1.265 | 0.942 | 1.342 | 77.4% | 17.9% | 231 | 20.6% |
| ACC 2021 | NA | NA | NA | NA | NA | NA | NA | NA | NA | 0 | 0.0% |
| ESC 2013 | 10.9% | 94.5% | 43.8% | 72.9% | 1.983 | 0.943 | 2.102 | 89.1% | 5.5% | 96 | 8.6% |
| ESC 2019 | NA | NA | NA | NA | NA | NA | NA | NA | NA | 0 | 0.0% |
| **male (n = 1653)** | | | | | | | | | | | |
| MPA model | 69.3% | 63.0% | 71.1% | 61.0% | 1.875 | 0.487 | 3.847 | 30.7% | 37.0% | 854 | 51.7% |
| ACC 2012 | 23.7% | 83.4% | 56.7% | 54.3% | 1.422 | 0.916 | 1.553 | 76.3% | 16.6% | 328 | 19.8% |
| ACC 2021 | NA | NA | NA | NA | NA | NA | NA | NA | NA | 0 | 0.0% |
| ESC 2013 | 12.0% | 94.8% | 56.3% | 65.7% | 2.294 | 0.929 | 2.470 | 88.0% | 5.2% | 137 | 8.3% |
| ESC 2019 | NA | NA | NA | NA | NA | NA | NA | NA | NA | 0 | 0.0% |
| **female (n = 764)** | | | | | | | | | | | |
| MPA model | 17.9% | 96.0% | 83.3% | 51.0% | 4.440 | 0.855 | 5.191 | 82.1% | 4.0% | 51 | 6.7% |
| ACC 2012 | 24.1% | 82.9% | 82.3% | 24.8% | 1.410 | 0.915 | 1.540 | 75.9% | 17.1% | 141 | 18.5% |
| ACC 2021 | NA | NA | NA | NA | NA | NA | NA | NA | NA | 0 | 0.0% |
| ESC 2013 | NA | NA | NA | NA | NA | NA | NA | NA | NA | 0 | 0.0% |
| ESC 2019 | NA | NA | NA | NA | NA | NA | NA | NA | NA | 0 | 0.0% |

Table indicates test characteristics of the MPA model and four commonly used pre-test probability scores. Cut-off was defined as very high risk (>85%). CAD: coronary artery disease. DOR: diagnostic odds ratio. FNR: false negative rate. FPR: false positive rate. NLR: negative likelihood ratio. NPV: negative predictive value. PLR: positive likelihood ratio. PPV: positive predictive value. PTP: pre-test probability.

Supplement Table S5: Definition of pre-test probability categories depending on MPA calibration

| **PTP category** |  | **MPA model** | **MPA model low risk** |
| --- | --- | --- | --- |
| Very low |  | 0 - 12 | 0 - 12 |
| Low |  | 12 - 32 | 12 - 71 |
| Medium |  | 32 - 73 | 71 - 87 |
| High |  | 73 - 82 | 87 - 92 |
| Very high |  | 82 - 100 | 92 - 100 |

The table indicates the numerical ranges of the pre-test probability category which differ between the two different MPA calibrations. PTP: pre-test probability.

Supplement Table S6: Comparison of the two different MPA calibrations

| **Predicted PTP** |  | **MPA model** | **MPA model low risk** |  | **Prevalence**  **Ischemia** |
| --- | --- | --- | --- | --- | --- |
| Very low |  | 4.0% | 4.0% |  | < 5% |
|  |  | (9.3%) | (9.3%) |  |  |
| Low |  | 7.7% | 22.3% |  | 5-15% |
|  |  | (8.6%) | (38.6%) |  |  |
| Medium |  | 27.6% | 46.1% |  | 15-50% |
|  |  | (32.0%) | (28.4%) |  |  |
| High |  | 36.7% | 53.0% |  | 50-85% |
|  |  | (12.6%) | (11.0%) |  |  |
| Very high |  | 60.4% | 72.9% |  | > 85% |
|  |  | (37.4%) | (12.7%) |  |  |

The table compares the distribution of patients within their estimated pre-test probability category between the original calibration^1^ and the low-risk population calibration^2^. The very low PTP category was identical, but discrimination in the low and medium PTP category was clearly better if the original MPA calibration was used in this patient cohort. The percentage indicates the prevalence of ischemia within each category and is color-coded according to the displayed values. Values in parentheses represent the percentage of patients in the corresponding category.

Supplement Figure S1: Patient flow


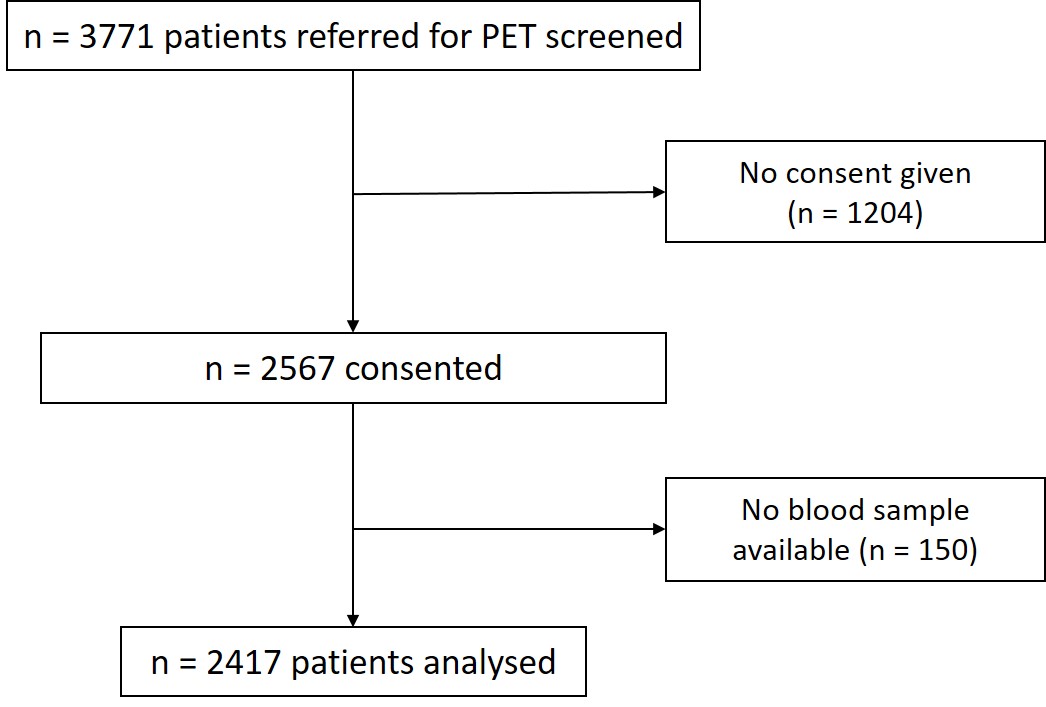


Figure illustrates the patient flow.

Reference Supplemental Material

1. Zellweger MJ, Tsirkin A, Vasilchenko V, et al. A new non-invasive diagnostic tool in coronary artery disease: artificial intelligence as an essential element of predictive, preventive, and personalized medicine. EPMA J 2018;9(3):235-247. DOI: 10.1007/s13167-018-0142-x.

2. Eurlings CGMJ, Bektas S, Sanders-van Wijk S, et al. Use of artificial intelligence to assess the risk of coronary artery disease without additional (non-invasive) testing: validation in a low-risk to intermediate-risk outpatient clinic cohort. BMJ Open 2022;12(9):e055170-e055170. DOI: 10.1136/bmjopen-2021-055170.
