# Supplementary material for: Artificial intelligence to improve ischemia prediction in Rubidium Positron Emission Tomography—a validation study
Source: EPMA J. 2023 Nov 15;14(4):631–43. doi: 10.1007/s13167-023-00341-5 (PMC10713509; doi:10.1007/s13167-023-00341-5)
Supplement: Supplementary file 2 — Supplementary file2 (DOCX 17.9 KB) [file 13167_2023_341_MOESM2_ESM.docx]

# PPPM Innovation Highlights

**Working hypothesis in the framework of PPPM**

Despite high clinical suspicion, a significant proportion of advanced cardiac tests for coronary artery disease (CAD) are normal (up to 60-70%). Hence, the patient preselection based on the current pre-test probability (PTP) tools as recommended by the guidelines are insufficient and need to be improved. Models using artificial intelligence (AI) can incorporate multiple, easily available variables and account for non-linear relationships and higher-order interactions between these. Hence, an AI model integrates these variables and is able to generate a more individualized profile of every patient. Therefore, we hypothesize in this study that AI could improve prediction of myocardial ischemia, help to preselect patients better prior to advanced cardiac testing and consequently personalize current diagnostic workflows.

To test our hypothesis, we analyzed a large cohort of patients referred for Rubidum Positron Emission Tomography (n = 2417). We tested whether a novel AI model predicts myocardial ischemia (surrogate for significant CAD) better than the PTP tools recommended by the current guidelines, and whether it improves allocation of patients across the whole spectrum of PTP.

**Innovation towards the**

**1. predictive approach,**

Whereas the current tools use three basic variables (symptoms, sex, age), the AI model offered more personalized patient assessment incorporating variables from clinical history, physical examination, ECG and biomarkers. It excelled the currently used PTP tools in terms of prediction of ischemia, allocation of patients across the PTP spectrum and excluding myocardial ischemia.

**2. targeted prevention and**

The AI model was the only tool to correctly identify patients with a very low PTP of ischemia. Hence, the MPA model could prevent patients without ischemia from unnecessary radiation and stress agent exposure. Furthermore, patients at risk according to the MPA model should be further evaluated and be referred for advanced cardiac testing.

**3. personalization of medical services**

Additionally, the AI model reduced the proportion of patients in the intermediate PTP range (15-85%) who do need an advanced cardiac test. Therefore, using the MPA model, the diagnostic pathway could be tailored to the individual patient. On one hand, this would include deferring patients without significant disease from cardiac tests. On the other hand, it would ensure that patients who benefit from advanced testing, will be tested. Hence, it is an excellent and safe gatekeeper candidate.

**How does the presented innovation go beyond the state of the art contributing to the paradigm shift from reactive medicine to predictive, preventive and personalized medicine (PPPM)?**

As described above, assessment of PTP and preselection of patients need to be improved. An increasing prevalence of cardiovascular risk factors and consequently CAD with limited health care resources warrants an optimal allocation of these resources.

The AI model improves this process in the direction of PPPM: First, analyzing multiple variables enables a more individualized, comprehensive and precise assessment of the prevalence of myocardial ischemia. This is important, because more precision is required to select patients who will profit from an advanced cardiac test. And on the other hand, protect patients, who will not profit from unnecessary radiation, by safely deferring them from the test.

Second, the model is based on variables which are readily available in every general practitioner’s practice. This offers early and personalized screening and identification of patients at much lower costs. This approach could also be extended to countries with less health care resources.

Third, the personalized assessment with this AI algorithm is promising and could be further extended to patient populations who are not yet symptomatic. This could help to identify patients at higher risk for CAD and start lifestyle interventions before the disease occurs.

Therefore, individualized PTP assessment with this AI tool shifts from the current “one fits all” diagnostic approach towards personalized precision medicine in line with the PPPM framework.
